# Ecological Change, Sliding Baselines and the Importance of Historical Data: Lessons from Combing Observational and Quantitative Data on a Temperate Reef Over 70 Years

**DOI:** 10.1371/journal.pone.0118581

**Published:** 2015-02-25

**Authors:** Giulia Gatti, Carlo Nike Bianchi, Valeriano Parravicini, Alessio Rovere, Andrea Peirano, Monica Montefalcone, Francesco Massa, Carla Morri

**Affiliations:** 1 DiSTAV, Department of the Earth, Environment and Life Sciences, University of Genoa, Corso Europa 26, Genoa, Italy; 2 CRIOBE, USR 3278 CNRS-EPHE-UPVD, LABEX ‘CORAIL’, University of Perpignan, Perpignan, France; 3 MARUM, University of Bremen, & ZMT, Leibniz Center for Tropical Marine Ecology, Leobener Str., Bremen, Germany; 4 Lamont-Doherty Earth Observatory, Columbia University, P.O. Box 1000, 61 Route 9W, Palisades, New York, United States of America; 5 ENEA, Marine Environment Research Centre, C.P. 224, La Spezia, Italy; Aristotle University of Thessaloniki, GREECE

## Abstract

Understanding the effects of environmental change on ecosystems requires the identification of baselines that may act as reference conditions. However, the continuous change of these references challenges our ability to define the true natural status of ecosystems. The so-called sliding baseline syndrome can be overcome through the analysis of quantitative time series, which are, however, extremely rare. Here we show how combining historical quantitative data with descriptive ‘naturalistic’ information arranged in a chronological chain allows highlighting long-term trends and can be used to inform present conservation schemes. We analysed the long-term change of a coralligenous reef, a marine habitat endemic to the Mediterranean Sea. The coralligenous assemblages of Mesco Reef (Ligurian Sea, NW Mediterranean) have been studied, although discontinuously, since 1937 thus making available both detailed descriptive information and scanty quantitative data: while the former was useful to understand the natural history of the ecosystem, the analysis of the latter was of paramount importance to provide a formal measure of change over time. Epibenthic assemblages remained comparatively stable until the 1990s, when species replacement, invasion by alien algae, and biotic homogenisation occurred within few years, leading to a new and completely different ecosystem state. The shift experienced by the coralligenous assemblages of Mesco Reef was probably induced by a combination of seawater warming and local human pressures, the latter mainly resulting in increased water turbidity; in turn, cumulative stress may have favoured the establishment of alien species. This study showed that the combined analysis of quantitative and descriptive historical data represent a precious knowledge to understand ecosystem trends over time and provide help to identify baselines for ecological management.

## Introduction

Marine coastal ecosystems are strongly affected by human activities, such as fishing, coastal development and pollution, which induce the compositional and functional change of communities [1]. Local human pressures are superimposed upon a changing climatic regime [2], whose major visible effect is the distributional shift of species according to their thermal tolerance and mass mortalities of vulnerable species [3].

Although crucial, the assessment of long-term ecosystems’ change is particularly challenging. Most measures of change, in fact, rely upon the comparison of present status to a defined baseline representing the reference condition. The “syndrome” of the sliding (or shifting) baselines describes the incremental lowering of ecological standards [4] and has become a major concern when assessing long-term change of ecosystems and planning conservation programs [5]. In absence of reliable historical information, the expectation of what the environment should look like depends on the individual scientist experience within his/her professional lifetime rather than on how the environment used to be in absence of human impacts. As a consequence, change may be measured using baselines that do not really represent a ‘pristine’ (or, at least, historical) condition. This, in turn, lessens the understanding of the ecosystem evolution, causes unreliable identification of pressures that produced the present status [6], and biases the estimation of the ecosystem services that went lost [7].

The challenge of delineating ecosystems’ change in the context of sliding baselines is particularly relevant because long quantitative time series are extremely rare, making the understanding of ongoing changes difficult and loosing important information for regional conservation plans. The need for defining reference conditions is more pronounced in highly populated areas, such as the coastal zones of the Mediterranean Sea, a semi-enclosed basin where human influence is so strong [8] that pristine areas do not exist anymore [9]. In such situations, the availability of historical data [10] assumes greater importance, but quantitative information is extremely rare [11]. On the other hand, descriptive information by early ecologists was collected most intensively along historically populated coastal areas [12]. These early datasets can be combined to more recent quantitative snapshots to understand long-term ecosystem change.

Coralligenous reefs are a deep biogenic habitat endemic to the Mediterranean, whose status urgently needs to be evaluated, [13]. Coralligenous reefs are shaped by the dynamic equilibrium between bioconstruction (encrusting red algae, with an accessory contribution by serpulid polychaetes, bryozoans and scleractinian corals) and destruction processes (borer species and physical abrasion); its assemblages are characterised by high biodiversity [14], biomass and production, almost comparable to coral reefs also in terms of calcification rate, around 10^3^ gCaCO_3_∙m^−2^∙y^−1^ [15]. Such reefs develop on both rocky and biodetritic bottoms from about 20 m down to 120 m depth, in dim light conditions and in relatively constant conditions of temperature, currents and salinity [16], and are sensitive to natural and human disturbances [17–18].

The large bathymetric distribution of coralligenous is the cause for sampling constraints due to operative limitations imposed by scuba diving [19]. As a consequence, coralligenous assemblages have been subjected to limited spatio-temporal investigations and historical information is seldom available [20]. Rapid non-destructive underwater protocols for the assessment of coralligenous status have been implemented only recently [21–23], so that comparable quantitative historical information is virtually unavailable.

In this study we analyse the oldest historical dataset existing in the Mediterranean Sea: the coralligenous assemblages of Mesco Reef (Ligurian Sea, NW Mediterranean). Decadal-scale change in the coralligenous assemblages has been assessed combining: i) a review of observational information since 1937; ii) the analysis of quantitative data since 1961; and iii) a descriptive reconstruction of major climatic and anthropogenic impacts acting in the area across the time frame considered.

## Material and Methods

### 2.1 Study area

**Fig 1:**
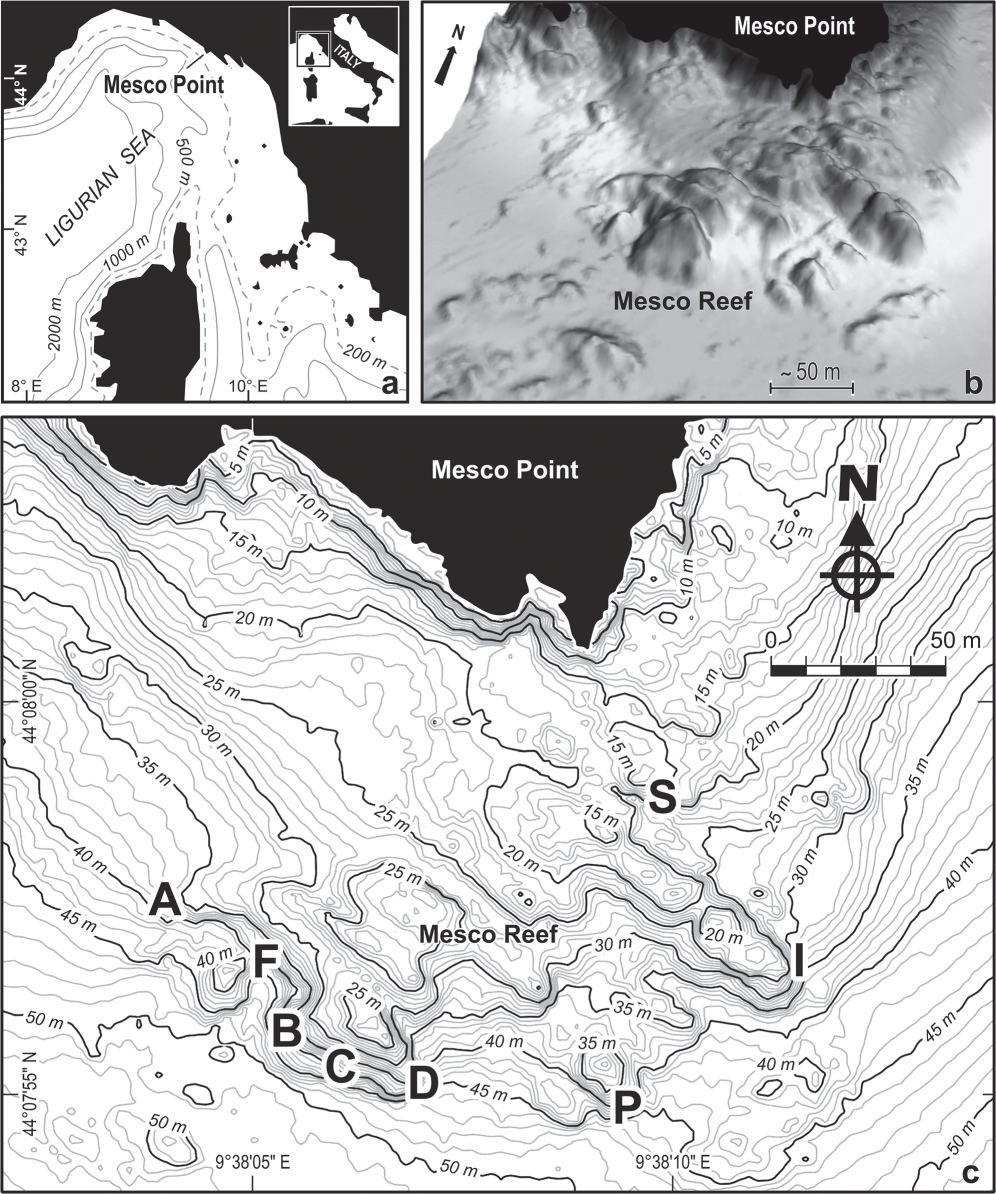
Study area. a) Geographical setting in the Ligurian Sea. b) Three-dimensional reconstruction of the Mesco Reef based on multibeam data from Regione Liguria. c) Bathymetric map of Mesco Reef, with study sites (capital letters).

Mesco Point is a rocky headland located along the eastern Ligurian coast (NW Mediterranean Sea), near the town of Monterosso al Mare (Fig. 1A). The headland points SE and is composed by sandstones (in contact with claystones and serpentinites) organized in strata, dipping SW as a plunging cliff. The rocky reef immediately off the point, for which only approximate descriptions existed [24–25], has been thoroughly investigated in 2008, using multibeam data and scuba surveys. Fieldwork have been authorised by the Cinque Terre Marine Protected Area authority and consisted in photographic surveys, without any direct manipulation of the organisms.

Three different groups of rocky outcrops can be recognised according to their depth: 1) outcrops at the cliff toe, between 8–12 m depth, which elevate 3–4 m on an irregular seabed made by rockfall and toppling deposits from the cliff; 2) outcrops at 15–25 m depth, which are 7–9 m high on coarse, partly biodetritic sand, with a relevant extension and a general NW-SE direction; 3) outcrops at 40–55 m depth, which elevate 2–5 m on fine, partly biodetritic sand.

A three-dimensional Digital Elevation Model (DEM), with a cell size of 0.25m, was extracted from the multibeam data (Fig. 1B). From the DEM, a bathymetric map of the study area (Fig. 1C) was derived using the *v*.*surf*.*rst*. command of GRASS GIS software. This command performs a spatial approximation and a topographic analysis using regularized spline curves to obtain a raster dataset from points with latitude, longitude and third dimension properties [26]. For a complete description of the script, the reader is referred to http://grass.osgeo.org/grass64/manuals/v.surf.rst.html.

### 2.2 Pressure regime

**Fig 2:**
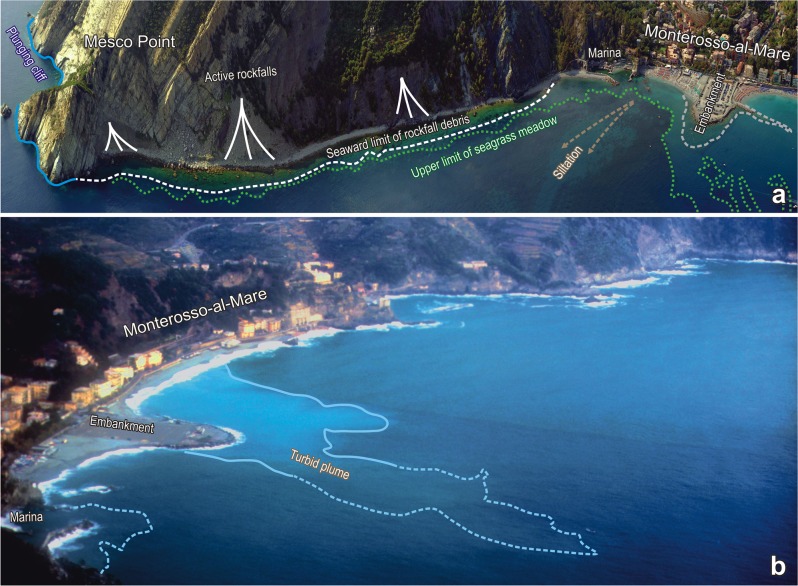
Mesco Point and its environments. a) Perspective photography (photo Regione Liguria) of the coastal tract between Mesco Point and the town of Monterosso-al-Mare, with the main nearshore geomorphological and ecological features. b) The beach of Monterosso-al-Mare viewed from the heights of Mesco Point (photo A. Peirano). The distance between Mesco Reef and the embankment is about 1400 m. Note the change in the structure of the embankment between the early 1990s (photo in b) and 2008 (photo in a).

Important environmental changes have occurred in the study area during the last decades (S1 Fig.). Since the 1970s, shelf waters have been warming, with a temperature increase of 1.1°C between the surface and 50 m depth and an acceleration during the 1990s [27]. A regime shift has been shown to occur at the end of the 1980s [28]. In addition to these large-scale climatic effects, which had important influence on the composition of the marine biota in the Ligurian Sea [29], also local human pressures increased in roughly the same decades. In the 1960s a small marina was built at the western extremity of the beach of Monterosso-al-Mare (about 1 km from Mesco Reef), whereas between 1963 and 1974 an embankment 150 m long and 90 m wide was created in the middle of the beach (Fig. 2), to serve as a car park during summer [30]. The beach underwent erosion since the construction of the embankment, which was reinforced with revetments in the 1980s and again in the 1990s; other static coastal defences (groynes, breakwaters, and seawalls) were also built [31]. Major beach replenishments have been done in the 2000s [32]. Coastal works have probably been responsible of the increased water turbidity in the area: water transparency (Secchi disk) passed from 23.8 m (± 1.3 se) in the 1950s to 11.8 m (± 0.9 se) in the 1990s [29] and to 12.7 m (± 0.3 se) in the 2000s [33]. Resident population decreased from 2000 inhabitants in 1960 to 1556 inhabitants in 2008 (data from the Italian Statistical Institute); on the contrary, tourism increased steadily to the current accommodation capacity of about 1200 people (data from the National Observatory of Tourism), which causes the population to nearly double during the summer. Since 1997, Mesco Point and Mesco Reef have been included in the “Cinque Terre” Marine Protected Area (MPA): as a consequence, fishing has been banned and diving strictly regulated.

### 2.3 Historical information on coralligenous assemblages

**Fig 3:**
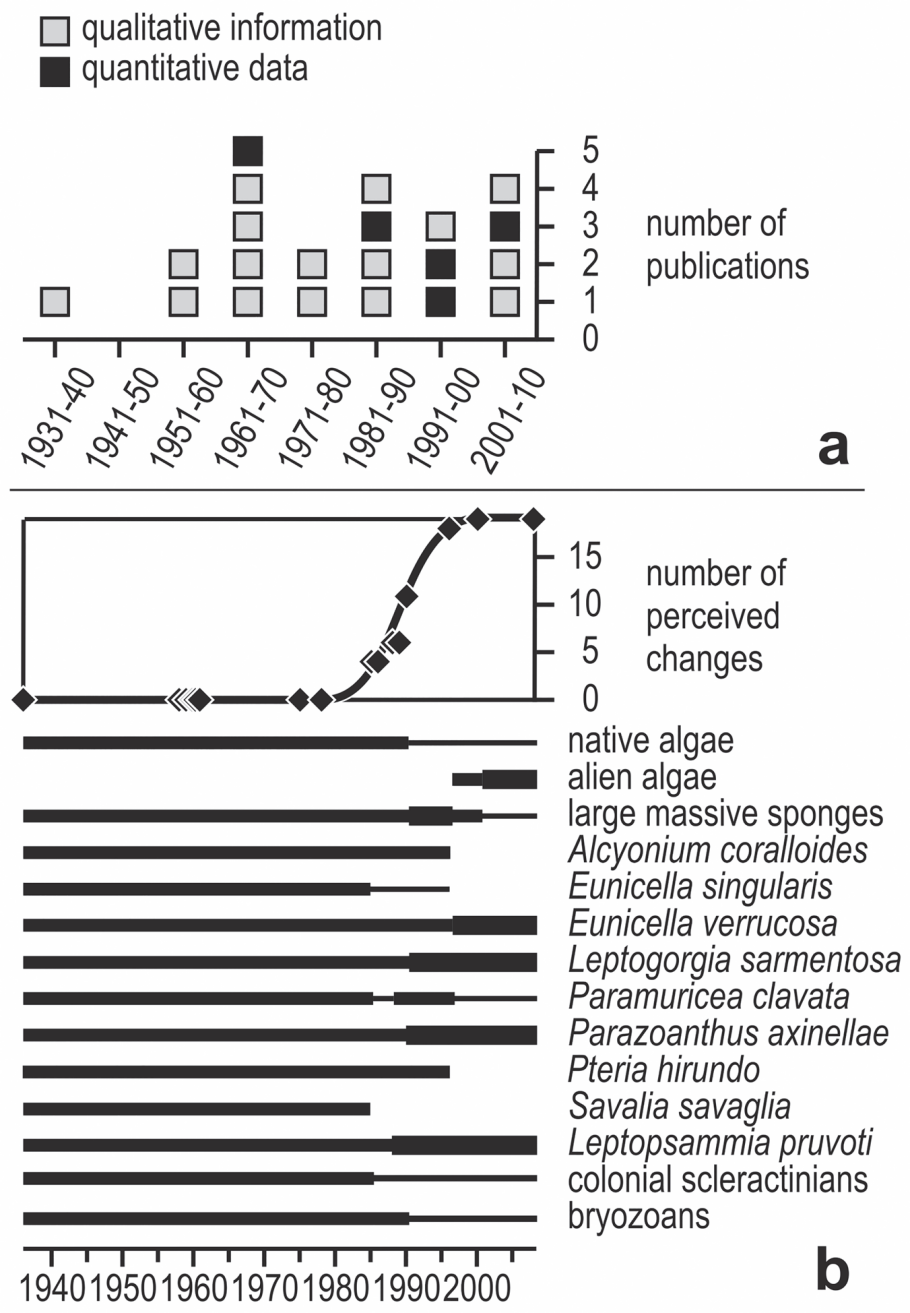
Data sources and historical analyses. a) Number of papers (each quadrat is one document) per decade on the coralligenous assemblages of Mesco Reef, according to the year of publication. Grey quadrats represent descriptive ‘natural history’ reports (including species lists and/or environmental information); black quadrats represent studies based on quantitative (cover) data. b) Change with time in the occurrence of a number of selected species mentioned in both qualitative and quantitative studies on the coralligenous assemblages of Mesco Reef. In the lower panel, continuous lines indicate the presumed persistence of a species, with thicker lines representing an increase in abundance or frequency and thinner lines a decrease, as perceived by the different authors. In the upper panel, diamonds represent the cumulative numbers of perceived changes, while the smoothed line indicates the general trend with time.

Starting from the 1930s, 21 papers (including grey literature and university theses) have reported on the coralligenous assemblages of Mesco Reef (Fig. 3A). One to five papers per decade are available, with the exception of the decade 1941–1950 (which encompasses the years of World War II and those of the economic crisis that followed), when no paper was published.

Many of the available publications provided descriptive ‘natural history’ reports (S1 Table), sometimes including precious species lists and/or environmental information [24],[25],[34–43].

The first quantitative data were collected in 1961 to 1962, when the diver Gianni Roghi shot underwater photos using quadrats of 1 m^2^ [25]. Roghi’s photoquadrats were later analysed by the biologist Lucia Rossi, who considered 25 images as workable to assess the percent cover of sessile conspicuous taxa [44]. Rossi described the species assemblages in relation to depth and slope of the substratum [45]. Based on published descriptions and the newly available bathymetric map and DEM, the photostations of Roghi [25] were relocated: the individual photoquadrats analysed by Rossi were grouped in 8 sites, individuated by the capital letters A, B, C, D, F, I, P and S (Fig. 1C, S2 Table). In 1988 [46], photoquadrats were used to collect quantitative data on anthozoans only. A study in 1990 [47] analysed all the sessile conspicuous species found in photoquadrats placed in 2 out of the 8 sites sampled by Roghi [25], namely S and I. In 1996, 51 photoquadrats (S2 Fig.) were sampled in 9 sites for a more complete survey of the sessile assemblages of the whole reef [48–50]; 7 of these sites were coincident with those used by Roghi [25]: B, C, D, F, I, P and S. Finally, 5 sites were sampled again in 2008 with photoquadrats, and all the sessile species were considered [51–52]: C, D, F, I and S. In the latter three studies, the percent cover of all the sessile epibenthic conspicuous organisms was obtained, as Rossi did [44]; voucher specimens of non protected species were collected only when necessary to identify problematic organisms, especially filamentous algae.

### 2.4 Data management and analysis

The qualitative information contained in all papers was first compared in a purely descriptive way [53]. Then, we focused on a number of selected species, mentioned by virtually all studies, paying attention at when each of them had been (subjectively) considered particularly abundant or frequent by the different authors. Unfortunately, unequivocal estimates of abundance were only seldom available, so that we have not been able to code these naturalists’ accounts as done for example in fishery [54]. However, the authors of a specific study often compared their observations with what had been already stated by earlier authors, explicitly underlying whether the perceived abundance or frequency of a given species showed increased or reduced. It has thus been possible to compute the number of perceived changes and illustrate them roughly as increases or decreases.

As regards quantitative information, species were classified on the basis of their change in cover over time as ‘losers’ if they decreased or disappeared between 1961 and 2008, or ‘winners’ if they conversely increased [55]. The category ‘commuters’ was introduced to represent those species that showed a major change in the 1990s, with no obvious difference between 1961 and 2008. Species that showed little or no change in cover over time were named ‘constants’.

A single data matrix (photoquadrats × time) × species was organised on the basis of available cover data for 1961–62 [44], 1990 [47], 1996 [48], and 2008 [51]. Data collected in 1988 on anthozoans only [46] were excluded. For each site and for each year, data from three photoquadrats, considered as site replicates, were compiled. Data on site A, available for 1961 only, were not included in the matrix because of lack of replicates and incomplete quantitative data.

Following Rossi [44], cover data were transformed into the following indices: 0.1 = cover negligible (< 0.5%); 1 = less than 5% of the surface; 2 = between 5% and 25% of the surface; 3 = between 25% and 50% of the surface; 4 = between 50% and 75% of the surface; 5 = more than 75% of the surface. Transformed cover data were submitted to Correspondence Analysis (CA) in order to detect distinct species assemblages and to delineate their time trajectories [56]. Significance of the axes (*p* < 0.05) was evaluated through the tables of Lebart [57].

The temporal rate of change of the whole coralligenous community of the rocky reef was assessed by computing the average (± se) Euclidean distance between the photoquadrats of a given year and the centroid of the photoquadrats of 1961–62. Euclidean distances where chosen as a measure of dissimilarity as they emphasize differences in the proportional species composition and account for joint-absence information [58], which is consistent with the trends observed for our species categories, i.e. winners, losers, commuters, and constants. The average (± se) Coefficient of Variation (CV) of Euclidean distances was then calculated for each year in order to appreciate change in the overall biodiversity among photoquadrats, lower CV values indicating biotic homogenisation. Finally, pie diagrams were used to illustrate the change in pattern of average cover dominance.

## Results

### 3.1 Qualitative data

The first available qualitative information on coralligenous assemblages of Mesco Reef dates back to the 1930s (S1 Table). The existence of the coralligenous reef, at that time called “coralline bottom”, was guessed on the basis of the gorgonian *Paramuricea clavata* and other characteristic species found among fishery discards. The spreading of SCUBA diving after World War II allowed for the first exploration of the reef between the late 1950s and the early 1960s: the biotic assemblages were described in detail, with special attention to the anthozoans, and UW photographs of the sessile macrobenthos were analysed. *P*. *clavata* was dominant, and other abundant species included the anthozoans *Alcyonium coralloides*, *Caryophyllia smithii*, *Eunicella singularis*, *E*. *verrucosa*, *Leptogorgia sarmentosa*, *Leptopsammia pruvoti*, *Rolandia coralloides* and *Savalia savaglia*, the bryozoans *Cellaria fistulosa*, *Smittina cervicornis* and *Pentapora fascialis*, and the bivalve *Pteria hirundo*. Renewed research in the late 1970s found no remarkable qualitative differences with respect to the previous studies but underlined the scarcity of sponges and the richness of bryozoans. On the contrary, a reconnaissance survey carried out in 1985, in view of the institution of the Cinque Terre MPA, reported the disappearance of *S*. *savaglia* and a decrease of *E*. *singularis* and *P*. *clavata*, while *C*. *fistulosa* and *P*. *hirundo* were not found again; waters were extremely turbid, especially at depth. A reduced number of scleractinian species was recorded in 1988, while other anthozoan species, namely *P*. *clavata*, *Parazoanthus axinellae* and *L*. *sarmentosa*, showed more abundant in 1990. In the photos of 1996, the algae *Lithophyllum stictaeforme*, *Flabellia petiolata* and *Peyssonnelia* sp., recorded in previous surveys, were not found again, while *L*. *pruvoti* increased; filamentous algae, including the alien *Womersleyella setacea*, appeared. Increased water turbidity and temperature with respect to the earliest studies were considered the main responsible for the changes observed. In the survey of 2008, *E*. *singularis* and massive sponges were not found again, whereas *P*. *clavata* decreased; the composition of bushy bryozoan species changed, *W*. *setacea* showed prominent, and another alien alga, *Caulerpa racemosa*, appeared.

In summary, most perceived changes were reported between 1985 and 1996 (Fig. 3B): in that period, *S*. *savaglia* and *A*. *coralloides* disappeared, while native algae, *E*. *singularis*, colonial scleractinian corals, and bryozoans got scarcer; after that period, *E*. *verrucosa*, *L*. *sarmentosa*, *P*. *axinellae* and *L*. *pruvoti* became more abundant, and alien algae appeared. The number of perceived changes should not be considered as an artefact due to a greater intensity of studies: only 5 studies (8 papers) between 1985 and 2008 remarked differences with earlier information, the remaining ones (mostly between 1958 and 1978) did not (S1 Table).

### 3.2 Quantitative data

A total of 53 sessile conspicuous species, belonging to seven higher taxa, was recognised on photoquadrats between 1961–62 and 2008 (Table 1). Sponges were the richest group (20 species), followed by cnidarians (13) and bryozoans (7); the remaining groups were less represented (rhodophytes with 5 species, chlorophytes and polychaetes with 3 species each, and tunicates with 2 species).

**Table 1:** Total list of the species found in the photoquadrats, ordered according to their codes as used in Fig. 5, and their time trend. A ‘winner’ is a species whose cover has increased between 1961 and 2008, vice versa for a ‘loser’; a ‘commuter’ showed a change in the 1990s, while for a ‘constant’ species little or no change in cover was observed.

Code	Species (higher taxon)	Time trend
*Aac*	*Acanthella acuta* (Porifera)	constant
*Aco*	*Alcyonium coralloides* (Cnidaria)	loser
*Ada*	*Axinella damicornis* (Porifera)	loser
*Aor*	*Agelas oroides* (Porifera)	winner
*Apu*	*Acrosymphyton purpuriferum* (Rhodophyta)	constant
*Ave*	*Axinella verrucosa* (Porifera)	winner
*Cce*	*Cliona celata* (Porifera)	winner
*Ccl*	*Clathrina clathrus* (Porifera)	commuter
*Ccr*	*Crambe crambe* (Porifera)	winner
*Ccy*	*Caulerpa cylindracea* (Chlorophyta)	winner
*Cfi*	*Cellaria fistulosa* (Bryozoa)	loser
*Cin*	*Caryophyllia inornata* (Cnidaria)	constant
*Cre*	*Chondrosia reniformis* (Porifera)	constant
*Dfr*	*Dysidea fragilis* (Porifera)	winner
*Esi*	*Eunicella singularis* (Cnidaria)	loser
*Eur*	*Eurypon* sp. (Porifera)	constant
*Eve*	*Eunicella verrucosa* (Cnidaria)	winner
*Fpe*	*Flabellia petiolata* (Chlorophyta)	commuter
*Hcr*	*Haliclona cratera* (Porifera)	winner
*Hpa*	*Halocynthia papillosa* (Tunicata)	constant
*Hro*	*Haliclona rosea* (Porifera)	commuter
*Htu*	*Halimeda tuna* (Chlorophyta)	winner
Hyd	large hydroids (Cnidaria)	commuter
*Ior*	*Ircinia oros* (Porifera)	loser
*Iva*	*Ircinia variabilis* (Porifera)	commuter
*Leu*	*Leucosolenia* sp. (Porifera)	loser
*Lpr*	*Leptopsammia pruvoti* (Cnidaria)	winner
*Lsa*	*Leptogorgia sarmentosa* (Cnidaria)	winner
*Lst*	*Lithophyllum stictaeforme* (Rhodophyta)	loser
*Msa*	*Microcosmus sabatieri* (Tunicata)	commuter
*Pam*	*Phyllangia americana mouchezii* (Cnidaria)	loser
*Pax*	*Parazoanthus axinellae* (Cnidaria)	constant
*Pcl*	*Paramuricea clavata* (Cnidaria)	loser
*Pfa*	*Pentapora fascialis* (Bryozoa)	commuter
*Pma*	*Paramuricea macrospina* (Cnidaria)	constant
*Pmu*	*Polycyathus muellerae* (Cnidaria)	constant
*Pru*	*Peyssonnelia rubra* (Rhodophyta)	commuter
*Psp*	*Pleraplysilla spinifera* (Porifera)	constant
*Psq*	*Peyssonelia squamaria* (Rhodophyta)	constant
*Pte*	*Phorbas tenacior* (Porifera)	commuter
*Ptu*	*Protula tubularia* (Polychaeta)	constant
*Rgr*	*Reteporella grimaldii* (Bryozoa)	winner
*Rvi*	*Reptadeonella violacea* (Bryozoa)	constant
*Sce*	*Smittina cervicornis* (Bryozoa)	commuter
*Scu*	*Spirastrella cunctatrix* (Porifera)	winner
*Sdy*	*Salmacina dysteri* (Polychaeta)	commuter
*Sfo*	*Sarcotragus foetidus* (Porifera)	commuter
*Slo*	*Schizoporella longirostris* (Bryozoa)	constant
*Sof*	*Spongia officinalis* (Porifera)	loser
*Ssa*	*Savalia savaglia* (Cnidaria)	loser
*Sve*	*Serpula vermicularis* (Polychaeta)	constant
*Tin*	*Turbicellepora incrassata* (Bryozoa)	constant
*Wse*	*Womersleyella setacea* (Rhodophyta)	winner

**Fig 4:**
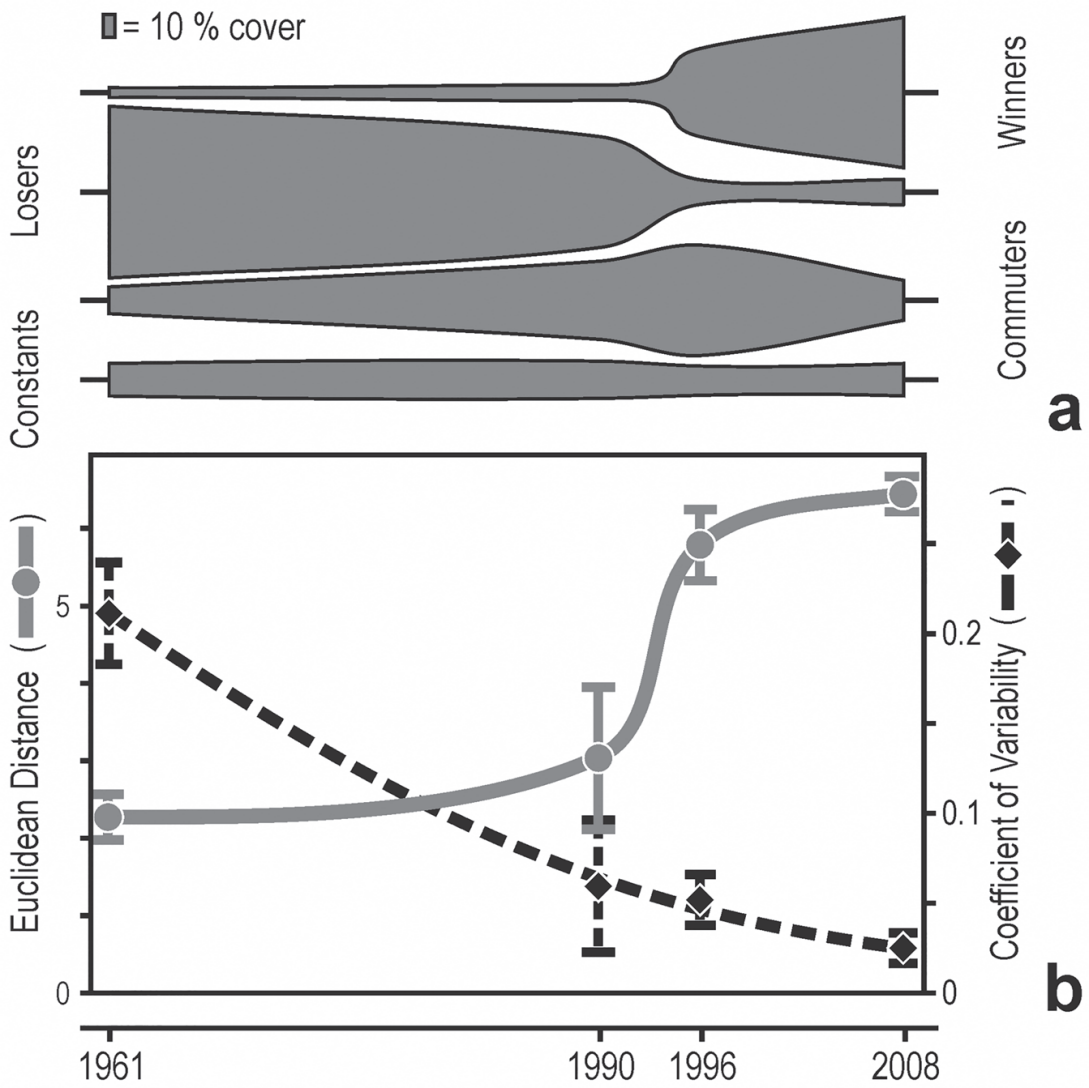
Quantitative historical analysis. a) Kite diagrams of the change in cover over time (as estimated from photoquadrats) of four categories of species: winners, losers, commuters, constants (see text). b) Average (± se) Euclidean distance among photoquadrats and their coefficient of variability from 1961 to 2008.

Based on their change in cover with time, 14 species were labelled as winners. Some of them (e.g., *Leptogorgia sarmentosa*) were already present in the photoquadrats of 1961–62, but most winners (e.g., *Womersleyella setacea*, *Spirastrella cunctatrix*, and *Eunicella verrucosa*) appeared for the first time in the photoquadrats of 1996 (S3 Fig.); *Caulerpa racemosa* was first observed in 2008: scarce within photoquadrats, this alien alga showed nevertheless already abundant in the area (S2 Fig.). Losers included 11 species, most of which disappeared (e.g. *Alcyonium coralloides*, *Savalia savaglia*, and *Cellaria fistulosa*) or reduced their cover (e.g., *Axinella damicornis* and *Lithophyllum stictaeforme*) in 1990 or 1996 (S3 Fig.). Commuters, whose cover underwent major change in1990 and/or 1996 (S3 Fig.), included 12 species (e.g., *Flabellia petiolata*, large hydroids, *Pentapora fascialis*, *Salmacina dysteri*, and *Sarcotragus foetidus*). Finally, 16 species showed constant, i.e., exhibited little of no change in cover on photoquadrats between 1961–62 and 2008 (Fig. 4A).

**Fig 5:**
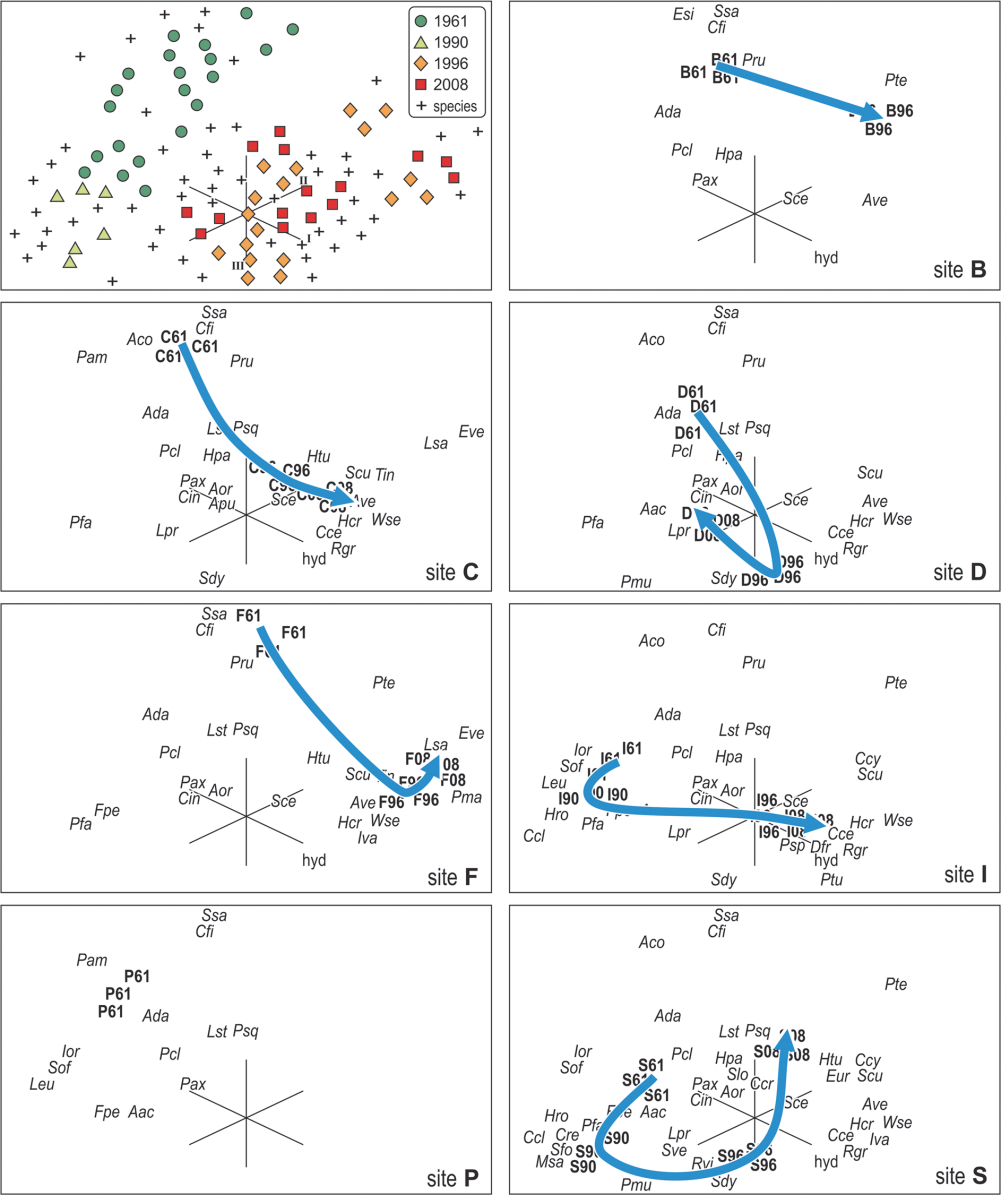
Ordination plot on the first three axes (Roman numerals) from Correspondence Analysis of the cover data matrix for the coralligenous assemblages of Mesco Reef, from 1961 to 2008. The upper left panel depicts the plot of all species points (crosses) and photoquadrat points (dots) to show the overall geometry of the ordination model. Details for each site are illustrated separately for the sake of clarity in the subsequent five panels, clockwise: trajectory and species of the assemblage at site B; trajectory and species of the assemblage at site C; trajectory and species of the assemblage at site D; trajectory and species of the assemblage at site F; trajectory and species of the assemblage at site I; position in 1961 and species of the assemblage at site P; trajectory and species of the assemblage at site S. Codes refer to the name of the species as showed in Table 1.

The first three axes of correspondence analysis were significant (Lebart’s tables, p < 0.05), explaining altogether 49.1% of the total variance (axis I: 21.9%; axis II: 15.7%; axis III: 11.5%). In the corresponding three-dimensional graph (Fig. 5), species-points disposed in two major clouds: the first, rather open, was located at the negative pole of axis I and included losers such as *Eunicella singularis*, *S*. *savaglia*, *C*. *fistulosa*, *A*. *coralloides* and *Phyllangia americana mouchezii* toward the negative pole of axis III, and commuters such as *Pentapora fascialis*, *Clathrina clathrus*, *Sarcotragus foetidus* and *Microcosmus sabatieri* toward the negative pole of axis II; the second cloud was denser, located at the centre and toward the positive poles of the three axes, and included *E*. *verrucosa*, *L*. *sarmentosa*, *C*. *racemosa*, *Axinella verrucosa*, *S*. *cunctatrix* and *W*. *setacea*. The site-points of 1961–62 and 1990 lay on the left of the three-dimensional graph, whereas site-points of 1996 and 2008 were intermixed with each other on the centre and toward the right. The time trajectories of the sites showed non-linear and, although each site had its own individual trajectory, all exhibited a more or less sharp deviation in 1990 and/or 1996, suggesting that change did not progress gradually.

The trend of the average Euclidean distance between photoquadrats indicated little change between 1961–62 and 1990, a sharp acceleration of the rate of change between 1990 and 1996, and again little change between 1996 and 2008 (Fig. 4B). On the contrary, the variability among assemblages (average Coefficient of Variation) decreased monotonously over time, describing a gradual process of biotic homogenization (Fig. 4B).

**Fig 6:**
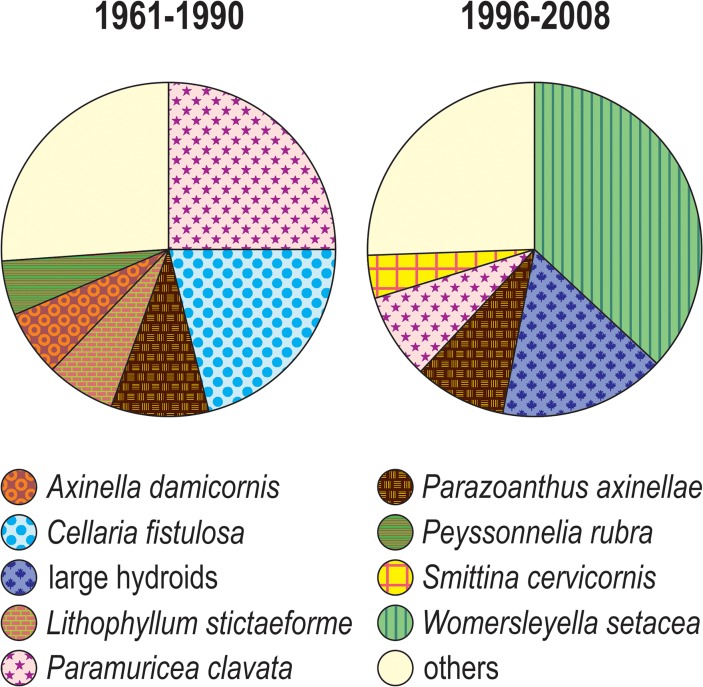
Pie diagrams of the average cover of the most important species for the periods 1961–1990 and 1996–2008, all sites confounded. Only the species with mean cover higher than 5% are considered, all the others are grouped within “others”.

All quantitative analyses were consistent in indicating that a major change occurred between 1990 and 1996. Until 1990, the coralligenous assemblages of Mesco Reef were on average dominated by gorgonians (*P*. *clavata*) and bryozoans (*C*. *fistulosa*); since 1996, the assemblages became dominated by filamentous algae (*W*. *setacea* among others) and large hydroids (Fig. 6).

## Discussion

The critical comparison of 21 studies and the analysis of 4 quantitative data sets (sessile species cover) allowed reconstructing the historical evolution of the coralligenous assemblages of Mesco Reef over 70 years. Most authors of those studies compared their observations with earlier descriptions, commenting upon the change that had occurred, if any. Although not immune to subjectivity, these perceptions of change showed a certain degree of consistence and in all suggested that major changes occurred between the late 1980s and the whole 1990s. These changes included the disappearance (or at least severe reduction) of formerly abundant species such as *Alcyonium coralloides*, *Eunicella singularis*, *Savalia savaglia*, and *Cellaria fistulosa*.

According to our analysis of quantitative data, the most dramatic changes in sessile species cover occurred during the 1990s, in agreement with the comparison of descriptive studies. Quantitative data suggested that a phase shift, i.e. a sharp change in community composition, took place between 1990 and 1996 in the coralligenous assemblages of Mesco Reef, which passed from a highly structured community having a dense canopy of large gorgonians (*Paramuricea clavata*), an understory dominated by long-lived calcified bryozoans (*C*. *fistulosa*) and a basal layer of encrusting corallines (*Lithophyllum stictaeforme*), to a homogenised community with a reduced *P*. *clavata*’s canopy, an understory with large hydroids and a basal layer of filamentous algae (dominated by the alien species *Womersleyella setacea*). Decreased abundance of calcified algae and increased abundance of aliens are consistent with the prediction of change for the whole NE Atlantic, although some ecosystems might of course show more stable than others [59].

Reduced canopy and scarcer calcified organisms at Mesco implied loss of structural complexity, with likely consequences on the whole associate community and ecosystem services [60]. The two alternative states showed comparatively stable for many (1961–1990) or several (1996–2008) years, while the transition between them has been abrupt (1990–1996) and marked by the quantitative exuberance of a number of species we called “commuters”. Boudouresque [61] described as “Riou effect” (from the name of an island near Marseilles, France) the outburst of generalist species at the transition between two distinct species assemblages along a spatial gradient. What we observed at Mesco Reef was apparently a Riou effect along a temporal gradient.

A phase shift normally results from a regime shift, i.e., a large-scale and long-lasting change in the nature, intensity, and/or frequency of the factors that govern the dynamics of the ecosystem [62]. A regime shift has been documented since the end of the 1980s, when the whole Mediterranean Sea underwent a major change that encompassed atmospheric, hydrological, and ecological systems [28]. The phase shift observed at Mesco Reef suggests a link between basin-wide and local changes in climate and direct human pressures, to which added the absolute novelty of the invasion by alien species.

The most obvious aspect of climate change has undoubtedly been the increase in water temperature. Indeed, a strong warming trend of surface water temperatures in the NW Mediterranean has been described since the second half of 1980s [63]. Warming has been considered as the main cause of mass mortality of gorgonians and other benthic organisms in the area. Although the best documented episodes were those of 1999 and 2003 [64–66], the first cases had already been observed in the early 1990s [67–68]: contextually, the cover of the gorgonian *P*. *clavata* strongly reduced at Mesco Reef. Increased storm intensity in NW Mediterranean is another aspect of climate change that caused mortality in fragile sessile invertebrates of the coralligenous, such as erect bryozoans, sponges, gorgonians, etc. [18],[69]. Among local human pressures, fishing has been claimed as an additional cause for *P*. *clavata* mortality in the 1990s in NW Mediterranean [68],[70], whereas at Mesco Reef the indiscriminate collection by scuba divers has been considered as the major responsible for the disappearance of *Savalia savaglia* [46], “the most beautiful object that a scuba diver can draw out from the sea” [35].

Another obvious driver of change at Mesco Reef has been water turbidity. Due to the general cyclonic surface circulation in the Ligurian Sea, the waters around Mesco Point are affected by the sediment load coming from the Magra River, whose mouth is located about 30 km eastward [71]: consistently, Mesco waters have always been turbid [38]. Coastal works since the late 1960s through the 1990s caused further reduction of water transparency [28],[33], which first allowed for the development of sciaphilic species (such as *Parazoanthus axinellae* and *Leptopsammia pruvoti*) in shallow water to the detriment of algae [49], and then for an increase of *Eunicella verrucosa* and *Leptogorgia sarmentosa*, two species considered as indicators of turbidity and fine sedimentation [72–73].

The interaction of multiple stressors is known to frequently produce synergistic effects on ecological communities [74]. The combined effect of climatic and human pressures putatively altered the coralligenous reef of Mesco Reef since the early 1990s. Stressed ecosystems are said to be more prone to the establishment of alien invasive species [75]. *Womersleyella setacea* and *Caulerpa cylindracea*, two of the “worst invasive species” in the Mediterranean Sea [76], were first recorded in the Ligurian Sea in the 1990s [77–78]: the former appeared at Mesco Reef in 1996, to become dominant in 2008; the latter was discovered in 2008, but subsequent observations (G. Gatti, unpublished data) suggested it was rapidly expanding. *W*. *setacea* is known to induce profound changes in the reef epifauna [79], and *C*. *cylindracea* has been documented to overgrow long-lived sessile invertebrates, such as massive sponges, gorgonians and scleractinian corals [80–82], and to outcompete encrusting corallinales, the main builders of the coralligenous reefs [83]. Both invaders have tropical origin, and their success is helped by Mediterranean Sea water warming [84]. The filamentous turf formed by *W*. *setacea* is favoured by high sedimentation rates, and in turn retains sediment [77]; turf facilitates colonisation by *C*. *cylindracea* [85].

Such a cascade of facilitation effects likely depicts a paroxysmal positive feedback that drove the assemblages of Mesco Point into an alternative state. Although some recovery from the warming-induced mass mortalities of the 1990s has been recorded for *Paramuricea clavata* in the area [86], the habitat alteration caused by the invasion of the alien species leaves little scope for a reversal of the phase shift experienced by the epibenthic community of Mesco Reef. The protection measures implemented by the Cinque Terre MPA might help the return of the species that used to characterise the coralligenous reef of Mesco Reef in historical times [87]; however, they will hardly eradicate the aliens. The similar phase shift already described for Ligurian seagrass meadows, which have been colonised by alien invaders in recent decades, has been considered almost irreversible [88].

There are two main lessons that can be learnt from the results of this study. The first is how to reconstruct the trajectory of an ecosystem in the absence of regular data series amenable to rigorous statistical analysis. In the marine environment, historical series mostly come from fisheries, while are rare for benthic communities [11]. In the case of coralligenous reefs, the few historical series span for 25 years at maximum [20]. We used multivariate analysis of historical quantitative data [89] together with qualitative observations arranged into a chronological chain of events [90], to reconstruct the ecological history of the coralligenous assemblage of Mesco Reef. Adopting approaches more typical of historical ecology may prove to be the key to understand change and to provide information for environmental management [91]. The accumulation of observations and data for over 70 years represents a precious heritage to be used for conservation purposes, after the establishment of the Cinque Terre MPA in 1997. As for other sites, many old descriptions of the marine habitats are often buried in publications and reports that are not readily available to the international scientific community [92]: we recommend MPA administrators and environmental managers to promote efforts aimed at resuming and reorganising such fundamental information.

The second lesson to learn is about identifying an adequate reference condition. Historical reconstructions such as the one presented here may help setting baselines [10]. The 1961–62 situation illustrated with detailed quantitative data [44] remained stable for the following 30 years and, although differences in methods prevent any formal comparison, was probably the same as the one described nearly 30 years before [34]. Of course it is impossible to affirm that those pioneer surveys really captured the pristine condition of Mesco Reef, but no evidence is available that something had already changed before. Approximately 60 years of stability in a time frame when climatic and human impacts were negligible is strongly suggestive that Rossi’s data may well serve as a baseline for the coralligenous assemblages of Mesco Reef. However, a better understanding about the present ecosystem status is required before adopting the situation of the 1960s as a reference condition for future management actions [93]. The assemblages existing in the early 1960s showed comparatively resistant to the increased local human pressures in the 1970s and 1980s and perhaps to the early signs of environmental change in the late 1980s, but changed abruptly in the 1990s when invasive alien species colonised the coralligenous reef. Thus, the 1990s have been the turning point between two distinct situations. The new assemblages, with many once characteristic species lost, reduced complexity, biotic homogenisation, and aliens dominating, remained stable for the subsequent decade. Only continued monitoring will help envisaging the possibility for a reversal of such a phase shift, but the establishment of the aliens makes the goal of returning to the reference condition unlikely to reach. Aliens shape an unprecedented ecosystem, and there is no going back for ecosystems [8]. ‘Target’ conditions [94] may sometime be preferable to reference conditions, when the latter are unreachable. For instance, management actions may be directed to maintain the present status (i.e., the one existing at the time when the Cinque Terre MPA was established in 1997), accepting that aliens are now naturalized, as happened on land [95]. Management actions may set ‘directional targets’ describing a desired trend of continuous improvement in status but where a final endpoint cannot be identified ― perhaps a reasonable choice in a fast changing marine environment.

## Supporting Information

S1 Fig Left panel—Multidecadal trends of annual mean sea surface temperature (SST) and air temperature in the Ligurian Sea. SST data have been derived from NOAA satellite time series (http://www.esrl.noaa.gov/psd/cgi-bin/data/timeseries/timeseries1.pl), corrected with the ENEA CRAM oceanographic historical data bank MOIS, Mediterranean Oceanographic Information System (http://www.santateresa.enea.it/wwwste/siamen/home.htm); air temperature data come from the Meteorological Observatory of the University of Genoa. (http://www.distav.unige.it/rsni/meteo_sito/main.htm). *Right panel*—Change in water transparency (Secchi disk depth). Data for the 1950s and the 1990s were taken from Morri and Bianchi [29], those for the 2000s from Attolini and Coppo [33].
(PDF)

S2 Fig Left upper and lower panels—Andrea Peirano shooting photographs in 1996 using a rigid spacer and a frame marked in centimetres (photo C.N. Bianchi). *Central panel*—An example of a photograph illustrating a sessile assemblage dominated by the purple gorgonian *Paramuricea clavata* (photo A. Peirano). *Right panel*—The alien green alga *Caulerpa cylindracea* monopolizes the substratum at 19 m depth, overgrowing a partially bleached colony of the native coral *Cladocora caespitosa*, on September 24^th^, 2008 (photo V. Parravicini).
(PDF)

S3 Fig Selected examples of species whose cover (mean + se) has more or less regularly decreased from 1961 to 2008 (the ‘losers’), increased from 1961 to 2008 (the ‘winners’), or exhibited major change in the 1990s (the ‘commuters’). (PDF)

S1 Table Available literature on the coralligenous assemblages of Mesco Reef, with a short synopsis of the most relevant information. (PDF)

S2 Table The photostations studied by Rossi [44], their physical features and the sites to which they have been assigned for the purposes of the present work. For the localisation of the sites see Fig. 1.
(PDF)
