# Correction: Ecological Change, Sliding Baselines and the Importance of Historical Data: Lessons from Combing Observational and Quantitative Data on a Temperate Reef Over 70 Years

**DOI:** 10.1371/journal.pone.0123268

**Published:** 2015-03-30

**Authors:** The PLOS ONE Staff

There is an error in the title of this paper: “Combing” should be “Combining.” The correct title is: Ecological Change, Sliding Baselines and the Importance of Historical Data: Lessons from Combining Observational and Quantitative Data on a Temperate Reef Over 70 Years.

The correct citation is: Gatti G, Bianchi CN, Parravicini V, Rovere A, Peirano A, Montefalcone M, et al. (2015) Ecological Change, Sliding Baselines and the Importance of Historical Data: Lessons from Combining Observational and Quantitative Data on a Temperate Reef Over 70 Years. PLoS ONE 10(2): e0118581. doi:10.1371/journal.pone.0118581

## References

[1] Gatti G, Bianchi CN, Parravicini V, Rovere A, Peirano A, Montefalcone M, et al (2015) Ecological Change, Sliding Baselines and the Importance of Historical Data: Lessons from Combing Observational and Quantitative Data on a Temperate Reef Over 70 Years. PLoS ONE 10(2): e0118581 doi: 10.1371/journal.pone.0118581 25714413 10.1371/journal.pone.0118581 PMC4340909

