# Supplementary material for: Ecological Change, Sliding Baselines and the Importance of Historical Data: Lessons from Combing Observational and Quantitative Data on a Temperate Reef Over 70 Years
Source: PLoS One. 2015 Feb 25;10(2):e0118581. doi: 10.1371/journal.pone.0118581 (PMC4340909; doi:10.1371/journal.pone.0118581)
Supplement: S1 Fig — SST data have been derived from NOAA satellite time series (http://www.esrl.noaa.gov/psd/cgi-bin/data/timeseries/timeseries1.pl), corrected with the ENEA CRAM oceanographic historical data bank MOIS, Mediterranean Oceanographic Information System (http://www.santateresa.enea.it/wwwste/siamen/home.htm); air temperature data come from the Meteorological Observatory of the University of Genoa. (http://www.distav.unige.it/rsni/meteo_sito/main.htm). Right panel—Change in water transparency (Secchi disk depth). Data for the 1950s and the 1990s were taken from Morri and Bianchi [29], those for the 2000s from Attolini and Coppo [33]. (PDF) [file pone.0118581.s001.pdf]

Supporting Information

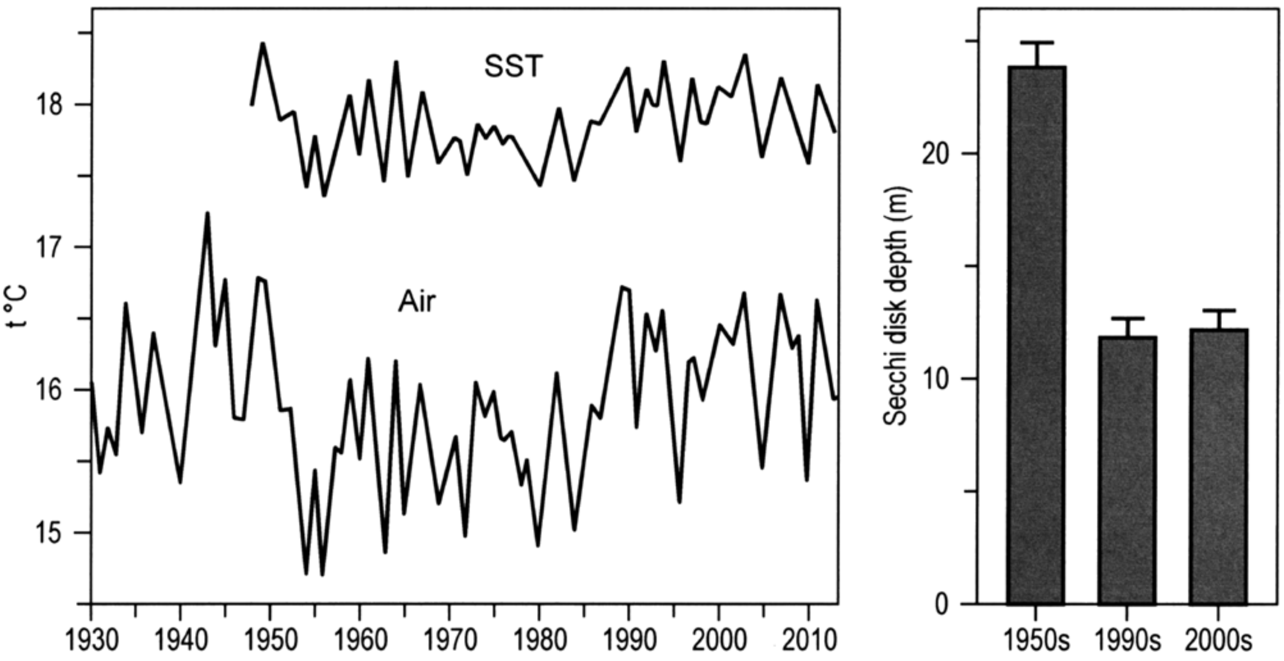

Figure S1. *Left panel* - Multidecadal trends of annual mean sea surface temperature (SST) and air temperature in the Ligurian Sea. SST data have been derived from NOAA satellite time series (www.esrl.noaa.gov/psd/cgi-bin/data/timeseries/timeseries1.pl), corrected with the ENEA CRAM oceanographic historical data bank MOIS, Mediterranean Oceanographic Information System (www.santateresa.enea.it/wwwste/siamen/home.htm); air temperature data come from the Meteorological Observatory of the University of Genoa (www.distav.unige.it/rsni/meteo_sito/main.htm). *Right panel* - Change in water transparency (Secchi disk depth). Data for the 1950s and the 1990s were taken from Morri and Bianchi [29], those for the 2000s from Attolini and Coppo [33].
