# Supplementary material for: Ecological Change, Sliding Baselines and the Importance of Historical Data: Lessons from Combing Observational and Quantitative Data on a Temperate Reef Over 70 Years
Source: PLoS One. 2015 Feb 25;10(2):e0118581. doi: 10.1371/journal.pone.0118581 (PMC4340909; doi:10.1371/journal.pone.0118581)
Supplement: S2 Fig — Central panel—An example of a photograph illustrating a sessile assemblage dominated by the purple gorgonian Paramuricea clavata (photo A. Peirano). Right panel—The alien green alga Caulerpa cylindracea monopolizes the substratum at 19 m depth, overgrowing a partially bleached colony of the native coral Cladocora caespitosa, on September 24th, 2008 (photo V. Parravicini). (PDF) [file pone.0118581.s002.pdf]

Supporting Information

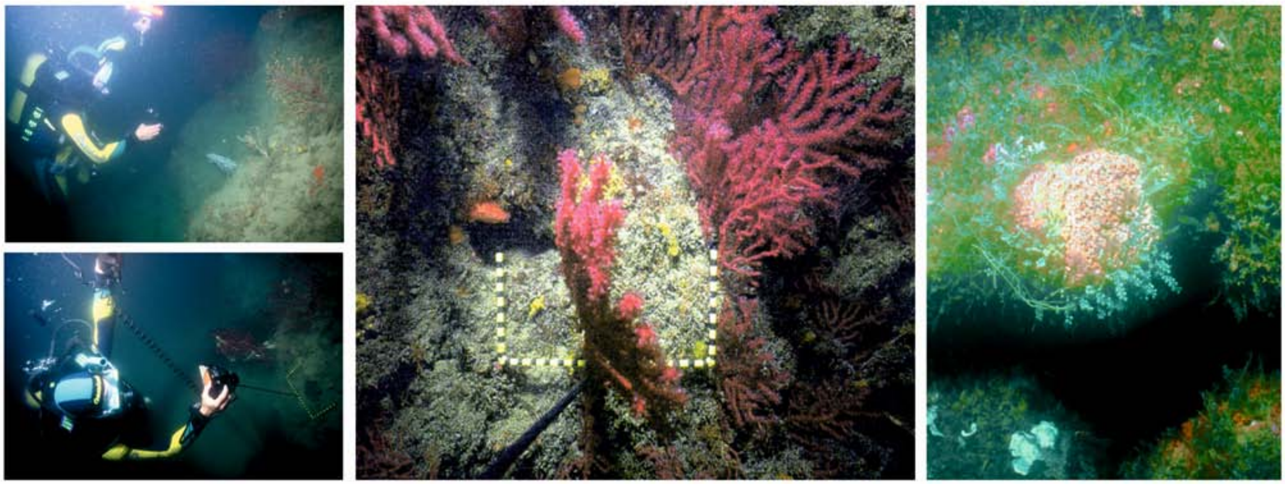

Figure S2. *Left upper and lower panels* - Andrea Peirano shooting photographs in 1996 using a rigid spacer and a frame marked in centimetres (photo C.N. Bianchi). *Central panel* - An example of a photograph illustrating a sessile assemblage dominated by the purple gorgonian *Paramuricea clavata* (photo A. Peirano). *Right panel* - The alien green alga *Caulerpa cylindracea* monopolizes the substratum at 19 m depth, overgrowing a partially bleached colony of the native coral *Cladocora caespitosa*, on September 24th, 2008 (photo V. Parravicini).
