# Supplementary material for: Ecological Change, Sliding Baselines and the Importance of Historical Data: Lessons from Combing Observational and Quantitative Data on a Temperate Reef Over 70 Years
Source: PLoS One. 2015 Feb 25;10(2):e0118581. doi: 10.1371/journal.pone.0118581 (PMC4340909; doi:10.1371/journal.pone.0118581)
Supplement: S3 Fig — (PDF) [file pone.0118581.s003.pdf]

Supporting Information

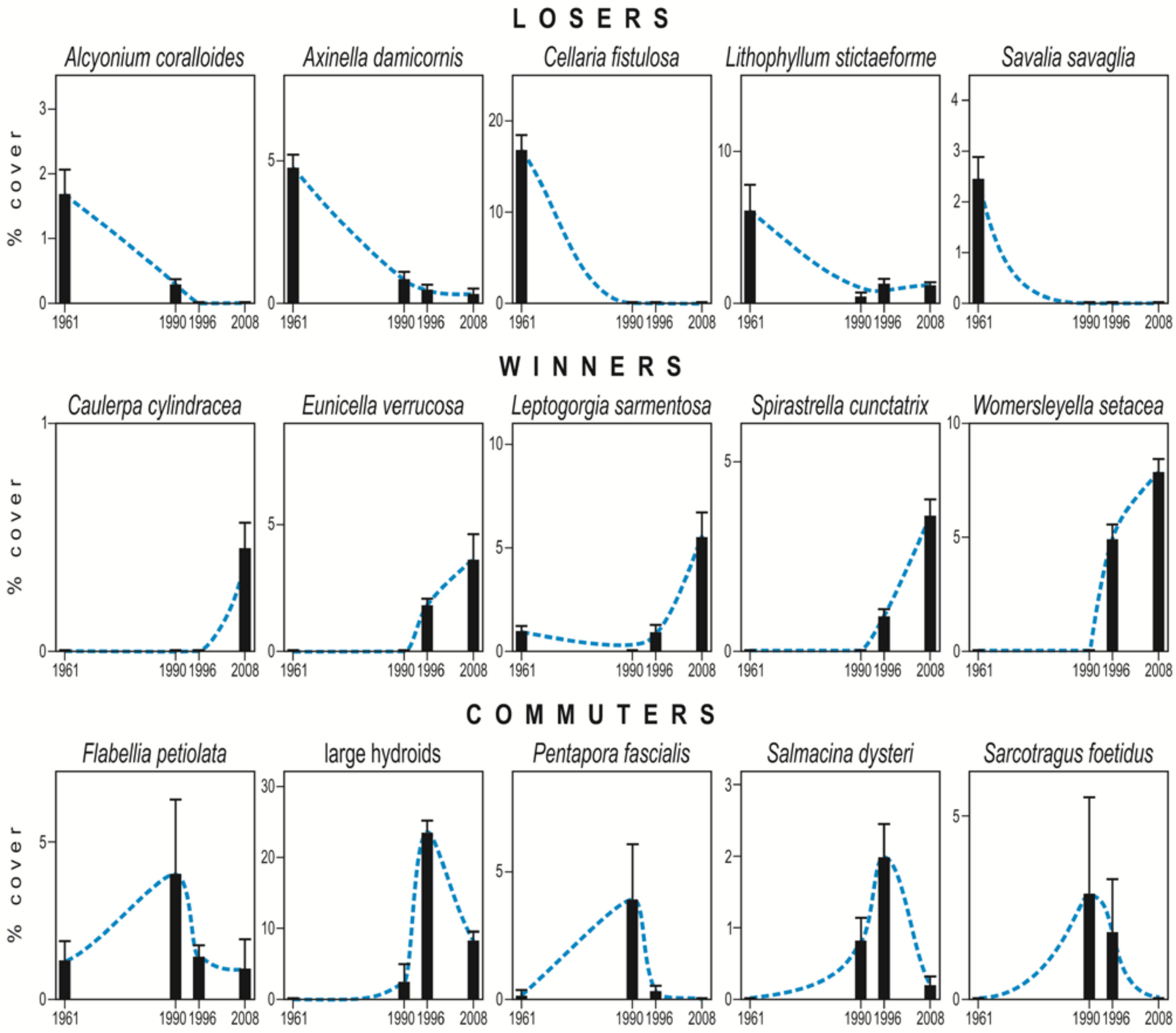

Figure S3. Selected examples of species whose cover (mean + se) has more or less regularly decreased from 1961 to 2008 (the 'losers'), increased from 1961 to 2008 (the 'winners'), or exhibited major change in the 1990s (the 'commuters').
