# Supplementary material for: Ecological Change, Sliding Baselines and the Importance of Historical Data: Lessons from Combing Observational and Quantitative Data on a Temperate Reef Over 70 Years
Source: PLoS One. 2015 Feb 25;10(2):e0118581. doi: 10.1371/journal.pone.0118581 (PMC4340909; doi:10.1371/journal.pone.0118581)
Supplement: S1 Table — (PDF) [file pone.0118581.s004.pdf]

Supporting Information

Table S1. Available literature on the coralligenous assemblages of Mesco Reef, with a short synopsis of the most relevant information.

Year	References	Information type	Main results
1936	Tortonese and Faraggiana [34]	Naturalistic observations.	Occurrence of *Petrosia dura* (= *P. ficiformis*), *Eunicella verrucosa*, *Paramuricea chamaleon* (= *P. clavata*) and *Retepora cellulosa* (= *Reteporella grimaldii*).
1958	Motta [24]	Underwater observations by scuba.	Seabed profile to 50 m depth, reef morphology, list of the most conspicuous species.
1958	Roghi [35]	Underwater observations by scuba.	Abundance and large size of *Gerardia savaglia* (= *Savalia s.*).
1959	Rossi [36]	List of species, qualitative information on octocorals.	Abundance of *Eunicella stricta* (= *E. singularis*) at 20-25 m depth; dominance of *Paramuricea chamaleon* (= *P. clavata*) at around 30 m depth, followed by *Leptogorgia sarmentosa*, *Eunicella verrucosa*, *Gerardia savaglia* (= *Savalia s.*), *Alcyonium coralloides* and *Rolandia coralloides*.
1960	Tortonese [37]	Naturalistic observations.	Abundance of *Eunicella stricta* (= *E. singularis*) in the 'precoralligenous', and of *Paramuricea chamaleon* (= *P. clavata*) and *Alcyonium coralloides* in the coralligenous; occurrence of *Gerardia savaglia* (= *Savalia s.*), *Eunicella verrucosa*, *Leptogorgia sarmentosa*, *Rolandia coralloides*, *Coenocyathus mouchezii* (= *Phyllangia americana m.*), and *Josephella marenzelleri*.
1960	Roghi [25]	Description of the underwater photographic survey methodology.	Abundance of *Paramuricea chamaleon* (= *P. clavata*) and *Gerardia savaglia* (= *Savalia s.*).
1961	Rossi [38]	Influence of environmental factors on biotic assemblages.	Fine sedimentation favours the occurrence of *Eunicella verrucosa*, *Alcyonium coralloides*, *Cellaria fistulosa*, *Pteria hirundo*; some bryozoans and scleractinian corals are mud-tolerant; presence of sciaphilic species at depths lower than usual due to water turbidity.
1961-62	Rossi [44]	Photoquadrats, cover data.	Eight biotic assemblages based on substrate slope and depth; high cover of gorgonacea, mainly *Paramuricea chamaleon* (= *P. clavata*), frequency of *Gerardia savaglia* (= *Savalia s.*).
1975	Associazione Parmasub [39]	Qualitative information, list of species.	No apparent pollution; little difference as compared to what previously reported by Rossi.
1978	Andreoli et al. [40]	Qualitative information.	Benthic assemblages in good conditions. *Paramuricea clavata* shallower than usual, sponges and scleractinian corals scarce, richness of bryozoans.
1985	Ardizzone and Belluscio [41]	ROV (Remotely Operated Vehicle) transects.	Short description of the coralligenous habitat as observed on videos. Presence of *Paramuricea clavata*, *Eunicella singularis*, *E. verrucosa* and *Lophogorgia sarmentosa* (= *Leptogorgia s.*).
1985	Relini et al. [42]	Qualitative descriptions.	Absence of *Gerardia savaglia* (= *Savalia s.*) and reduced frequency of *Eunicella singularis* and *Paramuricea clavata*, as compared to Rossi's observations; turbid water below 25 m and high sedimentation rate at depth.

1988	Peirano and Tunesi [46]	Photoquadrats, cover data on anthozoans.	Increased cover of *Paramuricea clavata* and *Leptopsammia pruvoti* as compared to Rossi's data. *L. pruvoti* also occurs in shallow waters. *Alcyonium coralloides* scarce.
1989	Tunesi et al. [43]	Visual survey, distribution of gorgonians.	Schematic distribution maps of *Eunicella singularis*, *Eunicella verrucosa*, *Paramuricea clavata* and *Leptogorgia ceratophyta* (= *L. sarmentosa*).
1990	Peirano and Sassarini [47]	Photoquadrats, cover data.	Dominance of *Paramuricea clavata*, *Parazoanthus axinellae* and *Leptogorgia sarmentosa*.
1996	Salvati [48], Peirano et al. [49], Bianchi et al. [50]	Photoquadrats, cover data.	Decreased algal cover and increased cover of *Parazoanthus axinellae* and *Leptopsammia pruvoti* in shallow water, as compared to Rossi's data, putatively due to increased water turbidity. *Alcyonium coralloides* and *Eunicella singularis* not recorded anymore.
2000	Morri and Bianchi [29]	Comparison of existing information on biotic cover and analysis of environmental data.	Great dissimilarity between biological data of the 1990s and the early 1960s; diminished water transparency between the 1950s and the 1990s; temperature increased between 1959 and 1996, much less so between 1990 and 1996.
2008	Roghi [51], Roghi et al. [52]	Photoquadrats, cover data.	Scarcity of massive sponges, change in bushy bryozoan species, and reduced gorgonian cover with respect to previous information; occurrence of the alien algae *Womersleyella setacea* and *Caulerpa racemosa* (= *C. cylindracea*).
