# Supplementary material for: Ecological Change, Sliding Baselines and the Importance of Historical Data: Lessons from Combing Observational and Quantitative Data on a Temperate Reef Over 70 Years
Source: PLoS One. 2015 Feb 25;10(2):e0118581. doi: 10.1371/journal.pone.0118581 (PMC4340909; doi:10.1371/journal.pone.0118581)
Supplement: S2 Table — For the localisation of the sites see Fig. 1. (PDF) [file pone.0118581.s005.pdf]

Supporting Information

Table S2. The photostations studied by Rossi [44], their physical features and the sites to which they have been assigned for the purposes of the present work. For the localisation of the sites see Figure 1.

Photostations	Description	Depth	Slope	Assigned to site
II	Rocks at the western end of the reef	40 m	40°	A
XI, XVI, XVII, XX	Flat to gently sloping rocks	38-42 m	< 30°	B
XII, XVIII, XIX	Rocks with medium inclination	38-44 m	45°-60°	C
V, XXII, XXIII	Rocks with steep inclination	40-44 m	65°-80°	D
IX, X, Xbis	Vertical and sub-vertical walls at shallow depths	17-23 m	70°-vertical	S
III, VI, VII	Vertical and sub-vertical walls at intermediate depths	30-35 m	70°-vertical	I
IV, VIII, XXIV	Deep vertical and sub-vertical walls	39-44 m	70°-vertical	P
XIII, XIV, XV, XXI	Deep depressions rich in sediment	40-43 m	low	F
